# Catalytic Behavior of Chromium Oxide Supported on Nanocasting-Prepared Mesoporous Alumina in Dehydrogenation of Propane

**DOI:** 10.3390/nano7090249

**Published:** 2017-09-01

**Authors:** Adam Węgrzyniak, Sebastian Jarczewski, Adam Węgrzynowicz, Barbara Michorczyk, Piotr Kuśtrowski, Piotr Michorczyk

**Affiliations:** 1 Institute of Organic Chemistry and Technology, Cracow University of Technology, Warszawska 24, 31-155 Kraków, Poland; wegrzyniak@indy.chemia.pk.edu.pl (A.W.); vinnicki@chemia.pk.edu.pl (A.W.); bmichorczyk@indy.chemia.pk.edu.pl (B.M.); 2 Faculty of Chemistry, Jagiellonian University, Gronostajowa 2, 30-387 Kraków, Poland; jarczewski@chemia.uj.edu.pl (S.J.); kustrows@chemia.uj.edu.pl (P.K.)

**Keywords:** nanocasting, chromium–aluminum catalysts, propane dehydrogenation, propylene

## Abstract

Mesoporous alumina with narrow pore size distribution centered in the range of 4.4–5.0 nm and with a specific surface area as high as 270 m^2^·g^−1^ was prepared via the nanocasting approach using a CMK-3 carbon replica as a hard template. Based on this support, a series of catalysts containing 1, 5, 10, 20 and 30 wt % of chromium was prepared by incipient wetness impregnation, characterized, and studied in the dehydrogenation of propane to propene (PDH). Cr species in three oxidation states—Cr(III), Cr(V) and Cr(VI)—were found on the oxidized surface of the catalysts. The concentration of these species varied with the total Cr loading. Temperature-programmed reduction (H_2_-TPR) and UV-Vis diffuse reflectance spectroscopy (UV-Vis-DRS) studies revealed that Cr(VI) species dominated at the lowest Cr content. An increase in the Cr loading resulted in an appearance of an increasing amount of Cr(III) oxide. UV-Vis-DRS measurements performed in situ during the PDH process showed that at the beginning of the catalytic test Cr(VI) species were reduced to Cr(III) redox species. A crucial role of the redox species in the PDH process over the catalysts with the low Cr content was confirmed. The stability test for the catalyst containing 20 wt % of Cr showed that this sample exhibited the reproducible catalytic performance after the first four regeneration–dehydrogenation cycles. Moreover, this catalyst had higher resistance on deactivation during the PDH process as compared to the reference catalyst with the same Cr loading, but was supported on commercially available alumina.

## 1. Introduction

Propylene can be obtained from propane by non-oxidative or oxidative dehydrogenation pathways [1,2,3,4]. The oxidative dehydrogenation of propane has not been commercialized yet, mainly due to problems with the control of selectivity with reasonable propane conversion (too much thermodynamically-favored CO*_x_* is usually formed). Some alternative processes, in which oxygen is replaced by weaker oxidizing agents (such as carbon dioxide), have been under investigation [3,4,5,6,7,8,9]. On the other hand, non-oxidative dehydrogenation of propane to propylene (PDH) is nowadays one of the most important forms of on-purpose technology for propylene production. Three type of PDH technologies—CATOFIN (Lummus Technology), Oleflex (UOP) and STAR (ThyssenKrupp Uhde)—have been developed on an industrial scale in more than 14 installations. Dozens of the new installations are already under construction or have been announced [3].

Despite the quite simple chemistry of PDH, the industrial implementation of this process is very complicated due to side reactions, such as cracking, polymerization, hydrogenolysis and coke formation. Important aspects in the PDH process are: (1) achievement of high per-pass conversion (near equilibrium conversion) and (2) limitation of side reactions and coke formation. Therefore, many efforts have continued to focus on the improvement of process configuration and catalyst formulation.

In commercialized PDH technologies, platinum- and chromium-based catalysts are widely applied [1,3,4]. Many other promising catalytic systems that are less hazardous for the environment than chromium or cheaper than platinum are being investigated, and are based on vanadium [10,11,12], gallium [13,14], iron [15,16], tungsten [17] or indium [18] oxides. In most cases, catalytically active compounds are deposited on supports characterized by a large specific surface area and accessible pore system. Moreover, promoters are added in order to enhance either selectivity or stability. For example, in the CATOFIN process, the *γ-*Al_2_O_3_-supported catalyst containing 18–20 wt % of CrO*_x_* is doped with 1–2 wt % of K or Na [3].

In the case of Cr-containing catalysts, many factors, including the type of precursors, Cr loading, and the preparation procedure as well as the support nature, influence the distribution of Cr species at various oxidation states, which has a direct impact on the catalytic performance in the PDH process [19,20,21,22,23,24,25,26]. In oxidized alumina-supported chromium oxide catalysts, chromium is stabilized in three oxidation states—Cr(III), Cr(V) and Cr(VI) [19,21,24]. During the dehydrogenation process these species form redox and non-redox Cr(III) sites. The former species arise due to the reduction of Cr(V) and Cr(VI) species in the initial period of the dehydrogenation process, while non-redox Cr(III) species originate from highly stabilized Cr(III) existing on the surface of the fresh catalyst [21,27,28]. It has been recognized that the presence of Cr(III) redox sites is crucial for high activity of the PDH catalyst [21,29,30]. The correlation between the number of redox Cr sites and activity is observed especially at low Cr loadings. At higher Cr contents, the impact of non-redox species on the catalytic activity cannot be neglected. Assuming the crucial role of Cr redox species in propane dehydrogenation, much attention has been paid to the selection of support with chemical properties and surface areas suitable for achieving a high contribution of redox Cr sites with a Cr loading as high as possible [31,32]. From this point of view, mesoporous supports, which offer high surface area and resistance to pore blocking (for instance by coke formed during catalytic runs), were found to be attractive. Very promising results were observed in the case of siliceous mesoporous materials with different pore architectures and surface areas [5,9,31,32]. For example, in propane dehydrogenation in the presence of CO_2_ we found that an increase in a specific surface area of silica by the application of mesoporous supports (SBA-15, SBA-1) increases the number of available Cr redox species and results in better PDH performance at high Cr loading [32]. 

In this work, we focused on the application of ordered mesoporous alumina instead of commercial alumina as a support for chromium oxide in a PDH catalyst. Various methods of synthesis of mesoporous alumina, with a surface area of 200–400 m^2^·g^−1^ and mesoporosity in the range 2–10 nm, were elaborated using different soft and hard templates, preparation procedures and precursors [33,34,35,36,37]. Herein, we used the nanocasting approach based on carbon hard templates (CMK-3) with the rod-type structure obtaining alumina with the hexagonal mesopore arrangement [35,37]. Using this alumina support we subsequently prepared catalysts containing 1–30 wt % of Cr_2_O_3_, which were characterized and tested in the dehydrogenation of propane to propene.

## 2. Results

### 2.1. Characterization of Alumina Support

The nanocasting route was applied for the preparation of pure Al_2_O_3_. Table 1 summarizes low-temperature nitrogen adsorption results for the hard templates (SBA-15 and CMK-3) as well as for the alumina support calcined at the various temperatures in air. The samples of alumina annealed between 600 and 800 °C are characterized by relatively high specific surface areas, which vary in the range of 250–270 m^2^·g^−1^, and narrow mesoporous-size distributions centered in the range of 4.4–5.0 nm (Figure S1). The thermal treatment at 900 °C brings about a significant reduction of specific surface area as well as broadening of the Barrett–Joyner–Halenda(BJH)pore size distribution. Moreover, in the samples calcined at 800 and 900 °C, the *γ*-Al_2_O_3_ phase appears (Figure S2). The phase transformation at high temperature (≥800 °C) is in a good agreement with results reported for mesoporous alumina prepared by the hard template approach [34]. Taking into account criteria of enhanced porosity and thermal stability under the conditions of the PDH process (typically 550–600 °C) a calcination temperature as high as 700 °C seems to be reasonable for the final thermal treatment of Al_2_O_3_-n. 

However, it should be noted that in comparison to SBA-15 (initial hard silica template), the Al_2_O_3_-n nanoreplicas have wider pore size distribution with a tail at larger pore sizes. Moreover, in the case of Al_2_O_3_-n nanoreplicas the maxima of pore size distribution and d_100_ values are lower than for SBA-15. For instance, in the case of Al_2_O_3_-n calcined at 700 °C the pore size maximum and the d_100_ values are 4.7 and 7.5 nm, and for SBA-15 these values are 8.1and 9.2 nm, respectively. Such changes of these parameters can be explained by the structural shrinkage process of carbon framework during the CMK-3 preparation as was reported previously [37].

**Figure 1:**
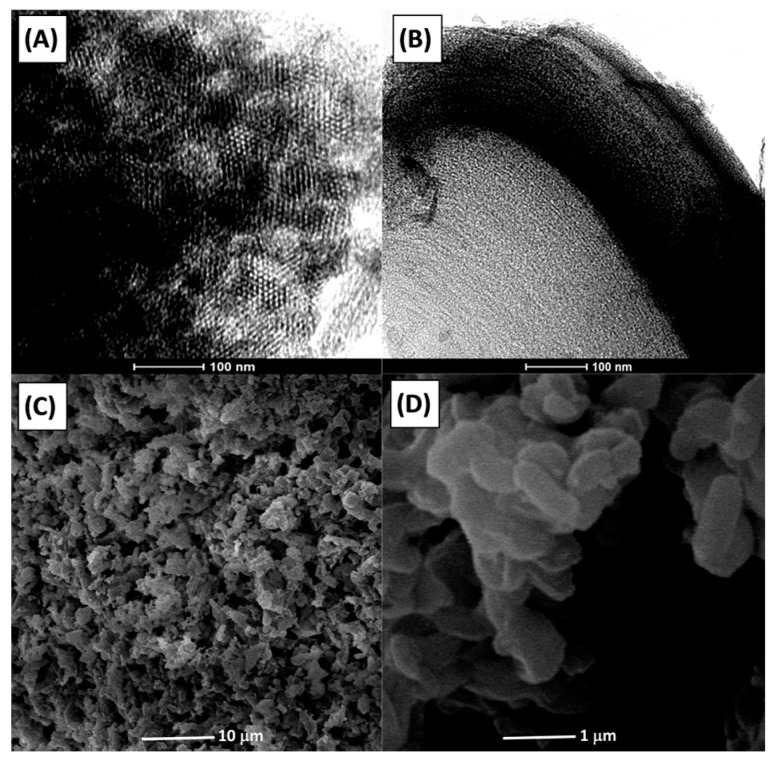
TEM (**A**,**B**) and SEM (**C**,**D**) images of Al_2_O_3_-n calcined at 700 °C.

Ordered pore structure and morphology of Al_2_O_3_-n calcined at 700 °C were confirmed by transmission electron microscopy (TEM) and scanning electron microscopy (SEM) analysis (Figure 1). As we reported previously, SBA-15 and its negative carbon replica (CMK-3) consist of wheat-like aggregates with rope-like domains characterized by a uniform size of about 1 μm [32]. 

Particles of Al_2_O_3_-n have similar shapes indicating that after replication the morphology of the initial rigid template is preserved (Figure 1C,D). The TEM micrographs (Figure 1A,B) indicate that the double replication results in a decrease of long-range structural order in comparison with the rigid templates (CMK-3 and SBA-15). 

### 2.2. Characterization of Catalysts

**Figure 2:**
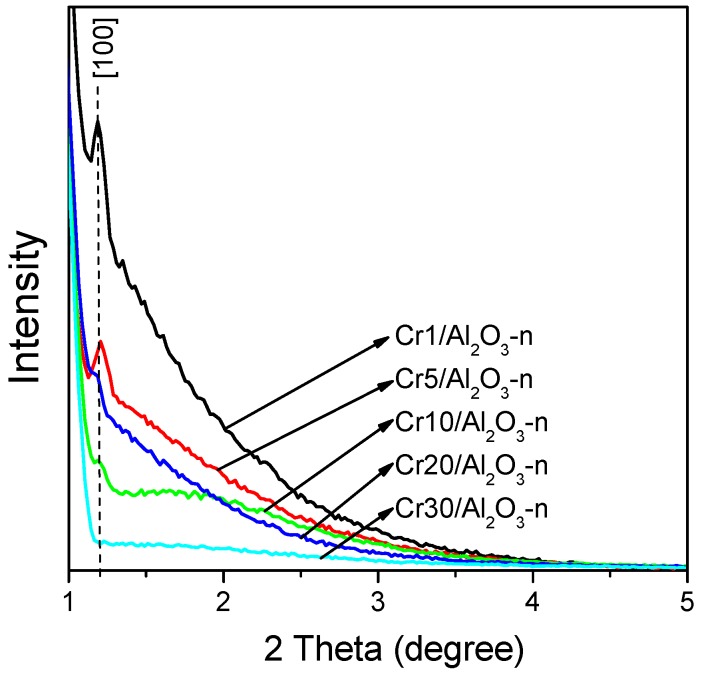
X-ray diffraction patterns of chromium oxide catalysts.

Based on ordered mesoporous alumina calcined at 700 °C, a series of Cr*x*/Al_2_O_3_-n catalysts were prepared with Cr loadings very close to those expected (Table 2). Figure 2 displays the X-ray diffraction (XRD) patterns collected for the calcined catalysts in the 2 theta range of 1–5°. The patterns of the samples containing between 1 and 20 wt % of Cr_2_O_3_ exhibit (100) reflection, which indicates the preservation of the hexagonal pore arrangement of the initial hard template (SBA-15) after repeated templating and impregnation. The intensity of the reflection decreases rapidly with the increase of the Cr content. The progressive reduction of the intensity of (100) reflection with the increase in the Cr loading is more likely due to a dilution of alumina by chromium introduced during impregnation as a consequence of its higher absorption factor for X-rays compared to alumina.

The maintenance of the ordered cubic arrangement of Al_2_O_3_-n frameworks upon deposition of chromium is further supported by the low-temperature N_2_ adsorption data. The isotherms and BJH mesopore distributions are displayed in Figure S3, whereas the textural parameters, such as specific surface area (*S*_BET_) and total pore volume (*V*_total_), are presented in Table 2. 

The *S*_BET_ and *V*_total_ decrease with the increase in the Cr_tot_ content, confirming a gradual filling of the pore system with chromium oxide. The deposition of 30 wt % of Cr_2_O_3_ results in a significant decrease in *S*_BET_ of about 50% in comparison to the parent Al_2_O_3_-n. Moreover, the Cr deposition also has an impact on pore size distribution (Figure S3). With the increase in the Cr content the maximum of pore size distribution shifts to lower values, that can be explained by the deposition of chromium species inside the pores.

**Figure 3:**
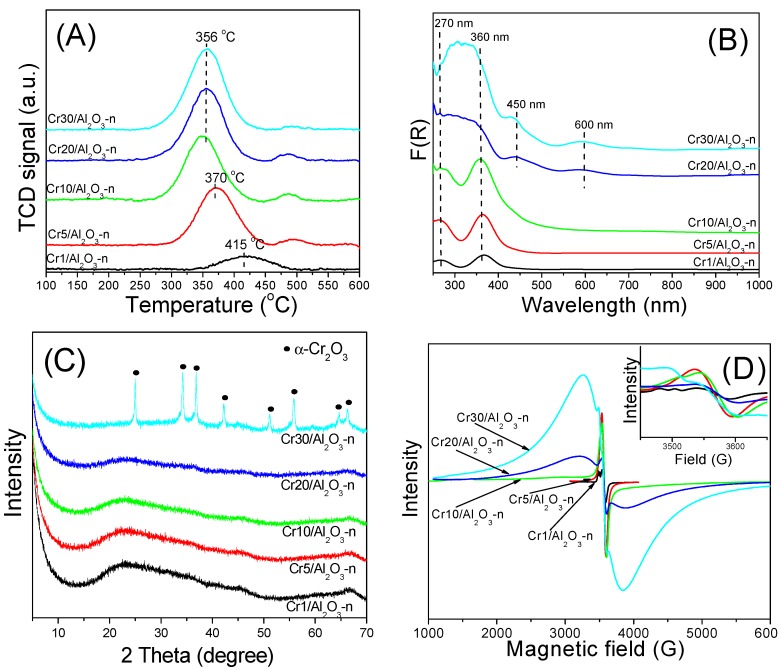
H_2_-TPR profiles (**A**); UV-Vis DRS spectra (**B**); XRD patterns (**C**); and electron paramagnetic resonance (EPR) spectra (**D**) measured for Cr*x*/Al_2_O_3_-n catalysts.

The oxidation state of Cr in the deposited species, reducibility and phase composition were subjected to careful investigation of the calcined Cr*x*/Al_2_O_3_-n catalysts by temperature-programmed reduction (H_2_-TPR), UV-Vis-DRS, XRD and electron paramagnetic resonance (EPR) (Figure 3). 

The H_2_-TPR profiles of all catalysts show only one reduction maximum in the temperature range from 356 to 415 °C (Figure 3A). In the case of chromium-alumina samples, this single peak related to hydrogen consumption is assigned to reduction of Cr(VI) to Cr(III) [20,21]. Its position shifts to lower temperature with an increase of the Cr content from 1 to 10 wt %, indicating changes in the dispersion of the Cr(VI) species. At higher Cr contents (>10 wt %), this maximum is found at the similar positions due to insignificant changes in the dispersion of Cr (VI) species. Table 2 summarizes the quantitative results of the H_2_-TPR measurements. The H_2_ consumption increases with the Cr loading up to 5 wt % of Cr_2_O_3_ and then only slightly for the catalysts with the higher Cr content, whereas the H_2_/Cr_tot_ ratio drops rapidly from 1.35 to 0.27 as the Cr loading is increased from 1 to 30 wt % of Cr_2_O_3_. In the case of the sample with the lowest Cr content, the H_2_/Cr_tot_ is close to the value of 1.5, which corresponds to theoretical reduction of Cr(VI) to Cr(III) species (2CrO_3_ + 3H_2_ = Cr_2_O_3_ + 3H_2_O). For higher Cr contents the H_2_/Cr_tot_ ratio is considerably lower. This indicates that above monolayer coverage of the support non-redox Cr sites are produced predominantly. A similar drop in the H_2_/Cr_tot_ ratio with the increase in the Cr loading was described for chromium oxide systems deposited on commercial alumina [21], titania [21] as well as on ordered and unordered mesoporous silicas [21,28,32].

The presence of Cr(VI) and Cr(III) species on the surface of the fresh catalysts confirms UV-Vis-DRS studies as well (Figure 3B). For all catalysts absorption bands at 270 and 360 nm are distinguished, which can be assigned to the O→Cr^6+^ charge transfer transitions in monochromate species. Specifically, the bands at 270 nm and 360 nm are due to the transitions ^1^A_1_→^1^T_2_ (1t_1_→7t_2_ and 6t_2_→2e) and ^1^A_1_→^1^T_2_ (1t_1_→2e), respectively [38,39].

Additionally, the spectra of the catalysts with the Cr content above 10 wt % show a shoulder about 450 nm and the broad band at 600 nm. The former band corresponds to either Cr_2_O_3_ or the dichromate/polymeric species (with low symmetry), while the band at 600 nm is attributed to d-d transitions of Cr^3+^ (^4^A_2g_→^4^T_2g_) in octahedral symmetry, as in Cr_2_O_3_ particles [38].

The formation of the Cr_2_O_3_ phase is also evidenced by XRD (Figure 3C). In the 2*θ* range of 10°–70°, the diffraction lines corresponding to the crystal phase of *α*-Cr_2_O_3_ are presented only in the XRD pattern of the sample with the highest Cr content (Cr30/Al_2_O_3_-n). The absence of the diffraction lines characteristic of the *α*-Cr_2_O_3_ phase in the XRD patterns of the catalysts with lower Cr loadings can be explained by high dispersion of this phase inside the mesopores of alumina support, which suppresses agglomeration during the preparation of the catalyst. We have previously reported very similar dispersion for materials based on mesoporous silicas, such as MCM-41, SBA-1 or SBA-15 [5,28,32]. 

The average crystallite size of the α-Cr_2_O_3_ phase detected in the Cr30/Al_2_O_3_-n catalyst was estimated using the Scherrer’s equation (D = (0.89 × *λ*)/(*β* × *θ*)), where: D—average crystallite size, 0.89—dimensionless shape factor, *λ*—X-ray wavelength, *β*—line broadening at half maximum intensity, and *θ*—diffraction angle. The estimated average crystallite size of *α*-Cr_2_O_3_ was equal to 32 nm, indicating that at very high Cr loading (30 wt %), presumably also larger crystals are formed outside the pore systems.

Figure 3D shows the EPR spectra of the Cr*x*/Al_2_O_3_-n catalysts. In the case of the studied chromium oxide materials, three main EPR signals are observed. The *γ*-signal with a linewidth of 3.5–6.0 mT at around g = 1.97 originates from the isolated Cr^5+^ species. The *β*-signal with a linewidth of about 78–85 mT and g = 1.99 is assigned to Cr^3+^ species in nearly octahedral coordination in small clusters. The third *δ*-signal, corresponding to the isolated Cr^3+^ species [39], consists of a broad line centered at about g = 1.9–2.2 and a positive lobe at about g = 2.5–4.5 [24]. For the Cr*x*/Al_2_O_3_-n catalysts, the *γ*-signal characteristic of Cr^5+^ species with the position of g = 1.97–1.98 and a linewidth in the range of 4–7 mT was observed. Moreover, in the samples with the Cr content ≥20 wt %, the additional broad *β*-signal originating from the agglomerated Cr^3+^ species appeared. For the same samples, the presence of UV-Vis DRS band at 600 nm confirms the EPR findings.

Changes in surface acidity after the Cr deposition on the Al_2_O_3_-n support were studied by temperature-programmed desorption of ammonia (NH_3_-TPD). The NH_3_-TPD profiles for Al_2_O_3_-n and the Cr*x*/Al_2_O_3_-n catalysts consist of a peak at ca. 170–180 °C followed by a peak with the maximum in the range of 290–310 °C and an overlapping peak at ca. 450 °C (Figure S4). The quantitative data on the acidity were obtained on the basis of mathematical deconvolution of the experimental TPD curves and by assigning the peaks with maxima at 190 °C to desorption of NH_3_ bonded by weak acid sites and those at 310 and 450 °C to the deliberation of medium–strong acid sites. The concentration of weak, medium–strong, and total number of acid sites normalized to the specific surface area are reported in Table 2. 

It is generally accepted that various alumina supports, including also mesoporous alumina, have solely Lewis acid sites [10]. As is shown the deposition of chromium has influence on both distribution and total concentration of acid sites. The concentration of strong-medium acid sites rises with the Cr content up to 20 wt % of Cr_2_O_3_, and then for the sample with the highest Cr content, it declines.

### 2.3. Catalytic Properties of Cr-Containing Samples

#### 2.3.1. Effect of the Nature of Cr 

First, the catalytic performance of the Cr*x*/Al_2_O_3_-n materials with different Cr loadings was examined in the PDH process. The initial specific activity, conversion of propane, yield of propene, as well as selectivity to propene, ethane, ethene and methane are summarized in Table 3, while variations of propane conversion and selectivity to propene vs. time-on-stream over the Cr*x*/Al_2_O_3_-n nanoreplicas are shown in Figure S5. To clarify, the initial catalytic performance of the chromium oxide-based catalysts (with Cr_2_O_3_ loading of 20 wt %) deposited on other mesoporous supports was reported as well. The Cr20/Al_2_O_3_-n catalyst exhibits better catalytic performance than Cr20/SBA-15 and Cr20/MCM-41, but lower performance in comparison to Cr20/CMK-3. However, in the latter case, the great limitation is catalyst regeneration because the CMK-3 support cannot be regenerated in an air flow.

It is clear that the conversion of propane and the yield of propylene achieved at 550 °C rise with the increase in the Cr loading up to 20 wt % and then decline. In this regard, the Cr*x*/Al_2_O_3_-n samples exhibit similar catalytic behavior to other Cr-containing materials deposited on oxide supports, such as SiO_2_ [32], SBA-15 [31,32], SBA-1 [9,28], ZrO_2_ [29], or Al_2_O_3_ [30]. Typically, over supported chromium oxide catalysts, prepared by impregnation, the catalytic activity in the dehydrogenation of hydrocarbons increases with a Cr loading reaching an optimal value. A further increase in the Cr content results in a decrease in the catalytic activity mainly due to pore clogging and/or formation *α*-Cr_2_O_3_ that is a less active phase [32]. The loading needed to achieve the maximum of catalytic performance changes in a wide ranges from 5 to 20 wt % and depends on synthesis conditions, kind of precursors and nature of support (textural parameters as well as phase and chemical composition). As we reported in previous works [28,32], over silica-supported chromium oxide catalyst activity in the PDH process correlates well with the number of redox Cr species in the fresh or regenerated catalysts. These sites are reduced during the initial step of PDH to catalytically active coordinatively unsaturated Cr species. 

**Figure 4:**
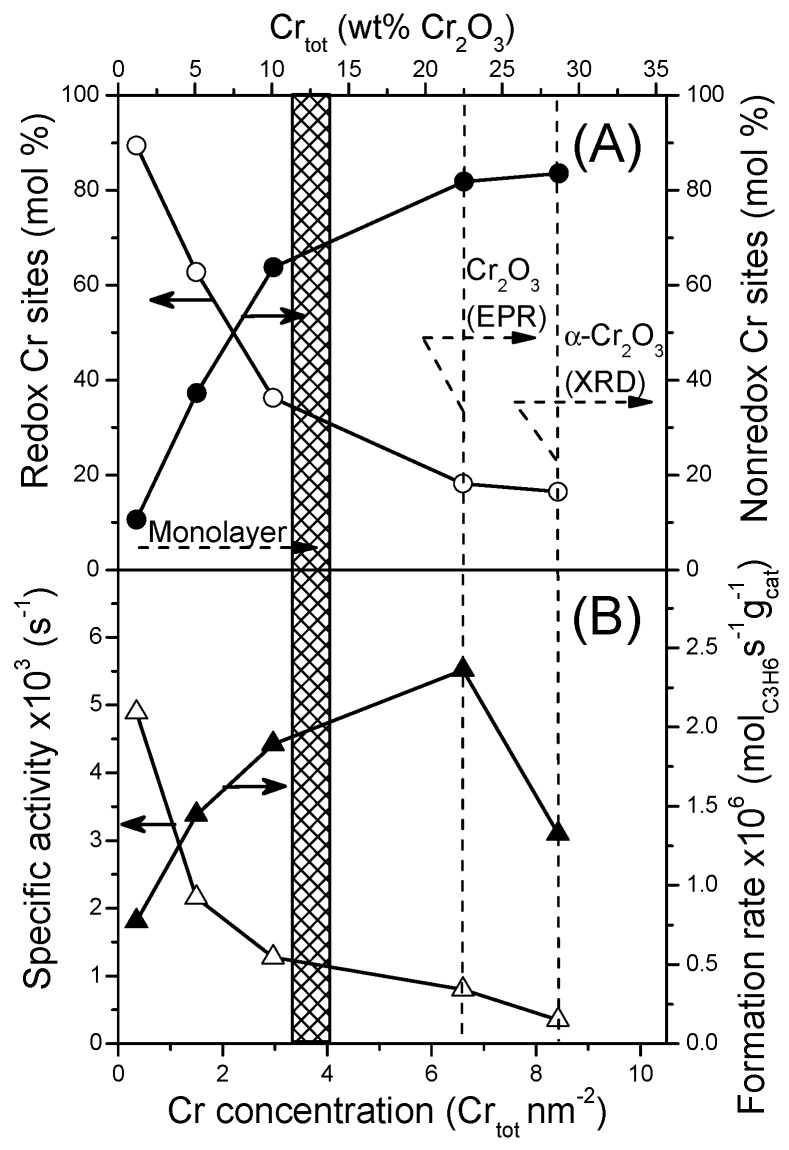
Variation of mole percent of redox and non-redox in origin Cr species (**A**) as well as specific activity and formation rate (**B**) with total Cr concentration. Reaction conditions: T = 550 °C; m_cat_. = 200 mg; Feed gas composition C_3_H_8_:He =1:14; Total flow rate = 30 cm^3^·min^−1^; WHSV = 1.2 h^−1^; Results collected after 10 min-on-stream.

Figure 4 illustrates variations of specific activity and rate of propylene formation in the PDH process as well as the percent of redox and non-redox in nature Cr sites with the total Cr concentration in the catalyst. The catalytic activity drops rapidly with an increase in the total Cr content (Figure 4B). A similar decrease is observed in mole percent of Cr redox species (Figure 4A) and confirms that this type of sites is crucial for high activity. The propylene formation rate normalized to 1 g of a catalyst rises almost proportionally with the Cr concentration below monolayer coverage that for an alumina support is between 3.7 and 4.5 Cr at. nm^−1^ according to different sources [30,40]. After exceeding the monolayer coverage, the formation rate increases slowly with a Cr loading. This effect can be related to the formation of small particles of Cr_2_O_3_ detected by UV-Vis DRS (band at 600 nm) and EPR (β-signal). The highest formation rate is achieved over the catalyst with 6.6 Cr at. nm^−1^ (20 wt % of Cr_2_O_3_). In this catalyst, the contribution of Cr redox species is only 18%. Above this concentration of Cr the formation rate declines rapidly. As was pointed above, the drop in activity correlates well with the appearance of *α*-Cr_2_O_3_ crystals located outside of the mesopore systems. These much larger crystallites present low catalytic activity.

Taking into account a contribution of redox Cr species in the catalytic activity, the changes in the oxidation state of Cr were studied during the PDH process by UV-Vis-DRS. The operando experiments were carried over two Cr*x*/Al_2_O_3_-n samples, e.g., Cr1/Al_2_O_3_-n enriched in the Cr redox species and Cr20/Al_2_O_3_-n, with a large number of non-redox Cr species.

**Figure 5:**
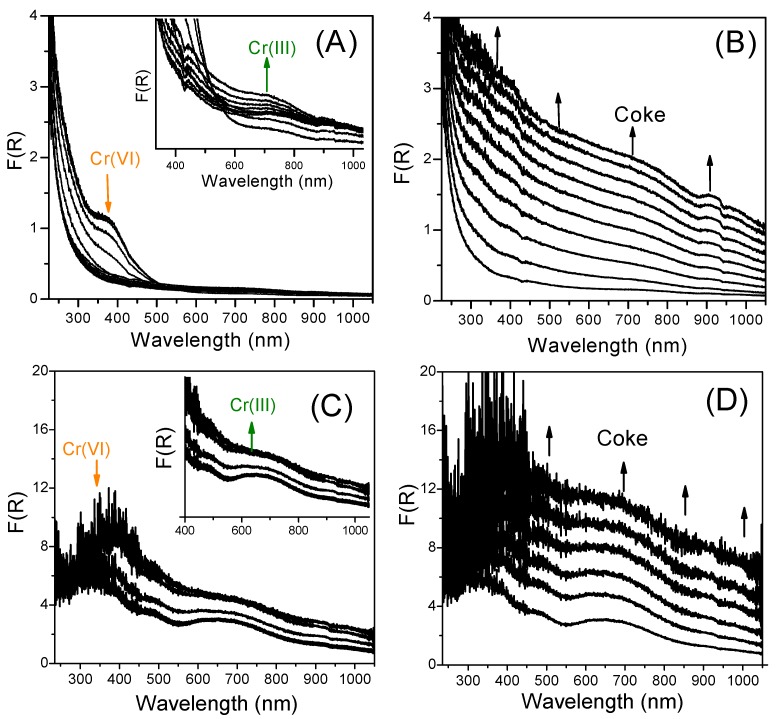
UV-Vis DRS spectra recorded during first 10 min (**A**,**C**) and between 10 and 240 min (**B**,**D**) of process over the Cr1/Al_2_O_3_-n and Cr20/Al_2_O_3_-n catalysts. Reaction conditions: T = 550 °C; m_cat._ = 200 mg; WHSV = 1.2 h^−1^; Feed gas composition C_3_H_8_:He =1:14; Total flow rate = 30 cm^3^·min^−1^.

Figure 5 shows UV-Vis-DR spectra recorded in the range of 250–1000 nm during the PDH process performed at 550 °C. For separation of the initial reduction and deactivation steps the spectra were divided into two ranges: (1) 0–10 min-on-stream, and (2) 10–240 min-on-stream. In the initial step of the PDH process, fast changes in intensity and shape of bands occur due to the reduction of Cr(VI) species. For both samples, the intensity of band in the CT range (about 360–380 nm) drops, while the new broad band appears at about 650–700 nm, evidencing the formation of Cr(III) species. However, in the case of Cr20/Al_2_O_3_-n, the tracking of Cr oxidation state is more complicated due to the high surface Cr concentration (weak signal in diffuse reflectance mode) and fast changes of the catalyst color (darkening) caused by carbon deposit. The deactivation of the catalysts by the coke can be more clearly observed between 10 and 240 min-on-stream (Figure 5B,D). In this period, the intensity of background, related to deactivation by coke, grows for both investigated catalysts.

It should be noted that in the case of alumina-supported chromium catalysts, the nature (redox and non-redox) and nucleation of the active Cr(III) sites are still subject of discussion [23,24,30,41]. The content of redox and non-redox Cr sites as well as their nucleation varies with preparation procedures, origin of alumina and Cr content, therefore the estimation of which of the Cr(III) sites exhibits the highest activity and the contribution in the PDH is difficult. In the case of our mesoporous catalysts, a good proportion between the number of redox sites and the catalytic performance in PDH is observed with the low Cr content where on the surface of the catalysts almost selectively redox species existed (89 mol %). However, the percent of redox species rapidly decreases with the Cr content indicating a rise in the content of non-redox Cr species contribution at the high Cr loadings. The letter species seem to play an important role in the catalysts containing 20 wt % of Cr_2_O_3_ over which the highest rate of propylene formation is observed.

#### 2.3.2. Effect of Support

An effect of nanocasting on the catalytic performance of the chromium oxide-based materials supported on Al_2_O_3_-n (calcined at 700 °C) obtained by nanoreplication and commercially available Al_2_O_3_-c was studied. Both the catalysts were prepared by an identical preparation procedure and contained the same intended amount of chromium (20 wt %), that was close to concentration of Cr in the industrial catalysts for the dehydrogenation of light alkanes used in the CATOFIN process [3]. 

Table S1 summarizes base characterization of pure Al_2_O_3_-c. The commercial Al_2_O_3_-c support has a lower specific surface area (S_BET_ = 161 m^2^·g^−1^) and porosity (V_tot_ = 0.24 cm^3^·g^−1^) than Al_2_O_3_-n (Table 1). Moreover, Al_2_O_3_-c is characterized by slightly higher total acidity in comparison with Al_2_O_3_-n and both supports have similar medium–strong acidity. Total and medium–strong acidity estimated based on NH_3_-TPD for pure Al_2_O_3_-c are 2.01 and 1.43 μmol NH_3_∙m^−2^, while in the case of Al_2_O_3_-n these values are 1.86 and 1.46 μmol NH_3_∙m^−2^, respectively. 

**Figure 6:**
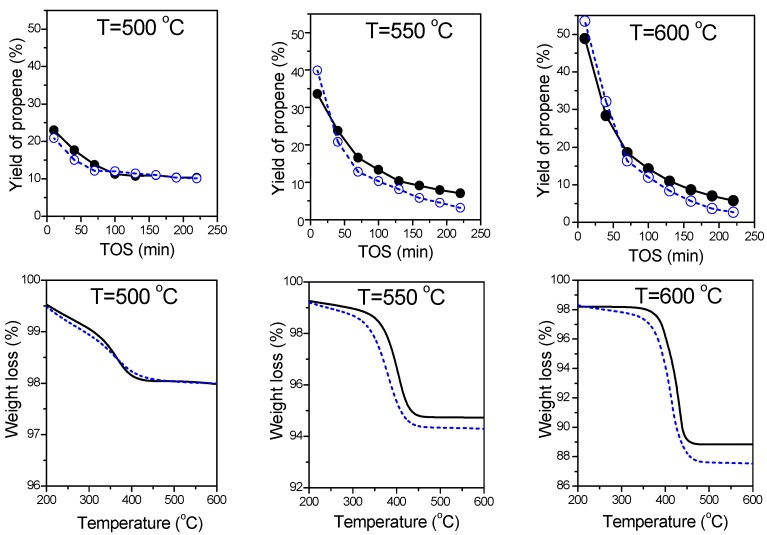
Comparison of Cr20/Al_2_O_3_-n (solid line) and Cr20/Al_2_O_3_-c (dot line) catalysts stability during PDH at 500. 550 and 600 °C. Reaction conditions: m_cat._ = 200 mg; Feed gas composition C_3_H_8_:He = 1:14; Total flow rate = 30 cm^3^·min^−1^; WHSV = 1.2 h^−1^.

Figure 6 shows variation of propene yield vs. time-on-stream (TOS) and thermogravimetric method (TG) profiles of used catalysts. At 500 °C the Cr20/Al_2_O_3_-c and Cr20/Al_2_O_3_-n catalysts exhibited a similar catalytic performance. A difference in the catalytic behavior between both investigated catalysts was revealed at higher temperatures. In the PDH process carried out at 550 and 600 °C the higher initial yield of propene was achieved over the Cr20/Al_2_O_3_-c catalyst. However, this catalyst deactivated faster in comparison with Cr20/Al_2_O_3_-n. After 30–60 min on-stream the yield of propene obtained over Cr20/Al_2_O_3_-n was higher at both studied temperatures. A comparison of the TG profiles of both catalysts reveals a difference in loss of weight only at 550 and 600 °C. In the mentioned temperatures higher loss of weight is observed in the case of Cr20/Al_2_O_3_-c indicating larger amount of deposited coke. Assuming that both alumina supports have similar medium–strong acidity, it can be proposed that the observed differences in the catalytic behavior and coke amount are related to porosity. A slower deactivation of the catalysts, based on the Al_2_O_3_-n nanoreplica, is probably connected with its higher specific surface area and ordered mesoporous arrangement forced by using the hard template.

#### 2.3.3. Regeneration Behavior

**Figure 7:**
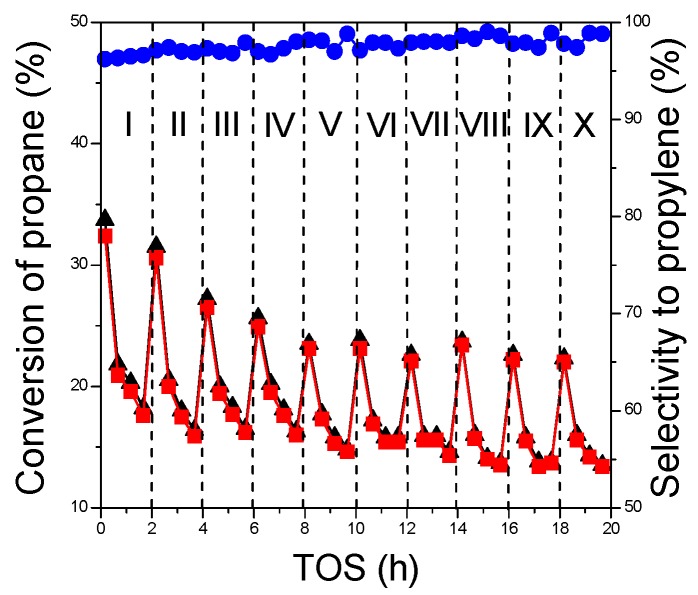
Variation of propane conversion (▲). propene yield (■) and selectivity (●) with time-on-stream in consecutive I–X cycles over Cr20/Al_2_O_3_-n. Dehydrogenation conditions: reaction temperature = 550 °C; catalyst weight = 200 mg; feed gas composition C_3_H_8_:He = 1:14; total flow rate = 30 cm^3^·min^−1^. Regeneration conditions: temperature = 550 °C; total flow rate of air = 30 cm^3^∙min^−1^. Purification conditions: temperature = 550 °C; total flow rate of helium = 30 cm^3^∙min^−1^. TOS: time-on-stream.

Finally, the stability of the Cr20/Al_2_O_3_-n catalyst in several dehydrogenation-regeneration cycles was studied. Figure 7 presents the variations in the conversion of propane, yield and selectivity to propene in the consecutive dehydrogenation steps. In each PDH step, the catalyst lost its activity due to the coke formation. 

After regeneration in the flow of air, the catalytic performance is partially restored. In the first four cycles the initial conversion of propane and the initial yield of propylene decreased by 3–5% in the consecutive PDH steps. A similar behavior of other PDH catalysts based on mesoporous supports, such as CrO*x*/SBA-1 or VO*x*/Al_2_O_3_, was discussed earlier [9,10,28]. Typically, the irreversible initial deactivation in the consecutive PDH cycles is caused either by structural changes of active species (such as agglomeration) or loss of regularity of mesoporous support.

In the case of the Cr20/Al_2_O_3_-n catalyst, after an initial period (four cycles) the catalytic performance in the further consecutive PDH cycles showed only insignificant differences, demonstrating good long-term stability.

## 3. Materials and Methods 

### 3.1. Catalyst Preparation

SBA-15 silica was synthesized in 2 M HCl solution using Pluronic P123 (EO20PO70EO20, Mav = 5800, Aldrich, St. Louis, MO, USA) as a template and tetraethylorthosilicate (98%, Aldrich, Shanghai, China) as a silica source. CMK-3 carbon was prepared using SBA-15 as a template and an aqueous solution of sucrose as a precursor of carbon [37,42].

Mesoporous Al_2_O_3_ support (denoted as Al_2_O_3_-n) was obtained by the incipient wetness impregnation method according to the procedure reported elsewhere [37]. In the typical synthesis, the CMK-3 was impregnated twice using a 1 M aqueous solution of Al(NO_3_)_3_ (99.96 wt %, Aldrich, Steinheim, Germany). In the first and second impregnations volumes of 4.0 and 3.2 cm^3^ of solution per 1 g of CMK-3 were used, respectively. After each impregnation step, the material was heated to 250 °C at a constant rate of 1 °C·min^−1^. Finally, the material was calcined in two steps. An inert gas (N_2_) atmosphere was obtained by raising the temperature from ambient-level to 600 °C at a rate of 1 °C·min^−1^, followed by maintenance of the final temperature for another 10 h. After cooling to room temperature, the sample was heated again in a flow of air using the same program, but with final temperatures of 600, 700, 800 and 900 °C, respectively.

Cr*x*/Al_2_O_3_-n catalysts were prepared by the incipient wetness method using Cr(NO_3_)_3_∙9H_2_O (99.6 wt %, Polish Chemical Reagents, Gliwice, Poland) as a chromium source. The support (1 g of Al_2_O_3_-n) was treated with 3.0 cm^3^ of an aqueous solution of Cr(NO_3_)_3_. The concentration of Cr(NO_3_)_3_ in solution was matched so as to obtain 1, 5, 10, 20 and 30 wt % of the total Cr_2_O_3_ content (Cr_tot_.) in the catalysts. The samples were dried overnight at room temperature, then kept at 60 °C for 6 h, and finally calcined at 600 °C for 6 h in a flow of air. The catalysts were denoted as Cr*x*/Al_2_O_3_-n, where *x* stands for nominal Cr_tot_ given in wt % of Cr_2_O_3_.

According to the same procedure, a reference catalyst was prepared using commercial γ-Al_2_O_3_ (Aldrich, Steinheim, Germany). Before impregnation, the support was calcined at 700 °C for 6 h in an air flow. The catalyst was prepared by impregnation of the alumina support with an aqueous solution of chromium nitrate (to obtain 20 wt % of Cr_2_O_3_ loading in the final samples) followed by thermal decomposition as in the case of the Cr*x*/Al_2_O_3_-n series. The catalyst was designated as Cr20/Al_2_O_3_-c.

### 3.2. Characterization Techniques

The total Cr content (Cr_tot_) in the samples (after mineralization) was determined using inductively-coupled plasma optical emission spectrometry (ICP-OES). An Optima 2100 DV spectrometer (Perkin-Elmer, Shelton, CT, USA) was used for the determination of Cr at the wavelength of *λ*_Cr_ = 267.716 nm. In the typical mineralization procedure, a sample (ca. 50 mg) was mixed with 2 g of NaNO_3_ (POCh-Polish Chemical Reagents, Gliwice, Poland) and 2 g of NaOH (POCh-Polish Chemical Reagents, Gliwice, Poland) in a melting pot. Then, the mixture was heated to 600 °C in air. The mineralization mixture was cooled to room temperature, dissolved in deionized water and filtered. Cr_tot_ was determined in a dissolved solution.

Temperature-programmed reduction (H_2_-TPR) and ammonia desorption (NH_3_-TPD) experiments were carried out in a modified gas chromatograph equipped with a thermal conductivity detector connected with a quartz reactor. In the TPR experiments, the mixture of N_2_/H_2_ (95/5 vol %, Air Liquide, Kraków, Poland) served as a combined carrier and reducing gas at a total flow rate of 30 cm^3^∙min^−1^. Before the H_2_-TPR experiments, the samples were dried at 120 °C for 12 h. The catalyst (50 mg) was preheated in the reactor in helium (99.999 vol %, Linde Gaz Polska, Kraków, Poland) at 600 °C for 30 min, and then cooled down to room temperature (RT) in the He stream. During the H_2_-TPR experiments, the temperature was raised from RT to 600 °C at the rate of 10 °C∙min^−1^. The H_2_ consumption was measured by thermal conductivity detector (TCD) after removal of water vapor by condensation. Ultra-pure NiO (99.999%, Aldrich) was used as a standard for calibration.

In the TPD experiments, a 100 mg sample was preheated in He at 600 °C for 2 h and then equilibrated with NH_3_ (99.98 vol.%, Linde Gaz Polska, Kraków, Poland) at room temperature. Physisorbed NH_3_ was removed by purging with pure He at 100 °C for 2 h. The TPD measurement was conducted by heating the sample from 100 to 700 °C at the rate of 10 °C min^−1^ in pure helium (30 cm^3^·min^−1^).

Low-temperature nitrogen adsorption–desorption isotherms were measured at −196 °C using a ASAP 2020 instrument (Micromeritics, Norcross, GA, USA). Before the measurements, the samples were degassed at 250 °C for 12 h. Specific surface areas (*S*_BET_) were calculated using the Brunauer–Emmett–Teller method within the relative pressure of *P/P*_0_ = 0.05–0.15. Pore size distributions were calculated from the desorption branches of the isotherms using the Barrett–Joyner–Halenda (BJH) model. Total pore volumes were obtained from the volumes of nitrogen adsorbed at *P/P*_0_ about 0.97.

XRD patterns were collected on a D2 Phaser diffractometer (Bruker, Madison, WI, USA) operated at 40 kV and 30 mA, equipped with a Cu K_α_ X-ray radiation (*λ* = 0.154 nm) with a step size of 0.02°.

Electron paramagnetic resonance (EPR) spectra were recorded at RT with a X-band Bruker ELEXSYS-580 spectrometer operating at 100 kHz field modulation with 1–5 G modulation amplitude. Morphology and structure of solids were investigated by means of a JEOL JSM-7500F field emission scanning electron microscope equipped with a transmission electron microscopy detector TED (total energy *detector*). In SEM experiments, catalysts were deposited on a sample holder. K575X Turbo Sputter Coater was used for coating the specimens with gold (deposited film thickness—20 nm). The specimens for the TEM study were prepared by depositing a small amount of powder samples on holey carbon films supported on copper grids.

Samples after a catalytic run in the PDH process were analyzed by thermogravimetric method (TG) in flowing air (100 cm^3^/min) using a SDT Q600 apparatus (TA Instruments, New Castle, DE, USA). A sample (ca. 5 mg) was placed in a crucible and heated to 900 °C at a rate of 20 °C/min.

### 3.3. Catalytic Tests

The catalytic performance of the synthesized samples was investigated in the dehydrogenation of propane to propene. The process was carried out in a tubular stainless-still reactor (SS316, i.d. 9.1 mm) packed with 200 mg of a catalyst (grain size 0.2–0.3 mm) in the temperature range of 500–600 °C and under atmospheric pressure using PID Microactivity-XS15. Before the PDH process, the sample was preheated at 600 °C for 30 min in an air flow and then for another 30 min in a dry He stream. After that, the reaction temperature was set and after stabilization time an inert gas (He) was replaced with a mixture of propane (99.96% Linde) diluted with He (99.9996% Linde, Gaz Polska, Kraków, Poland) 2/28 *vol.*/*vol*. at the total flow rate of 30 cm^3^∙min^−1^ (WHSV = 1.2 h^−1^). The products and unreacted substrates were analyzed using an Agilent 6890N gas chromatograph (equipped with Hayesep Q and 13X molecular sieves packed columns and TCD), connected with the reactor. Specific activity, rate of propene formation (activity), conversion of propane, yield of propene and selectivity to all hydrocarbon products were calculated based on carbon balance in inlet and outlet of the microreactor using the equations reported elsewhere [42].

### 3.4. Operando UV-Vis DRS

During selected PDH tests, the oxidation state of chromium species on the surface of alumina was monitored by operando UV-Vis-DR spectroscopy using Ocean Optics HR2000+ (integration time 20 msec, 20 scans, Winter Park, FL, USA) equipped with an Ocean Optics DH-2000 BAL (Ostfildern, German) halogen-deuterium light source and a FCR-7UV400-2-ME-HTX reflection probe (7 × 400 μm fibers, Anglia Instrument Ltd, Cambridgeshire, UK). The spectra were collected in the wavelength range of 225–1100 nm using BaSO_4_ as a reflection standard. The spectra are shown in the Kubelka–Munk format (F(R) = (1 − R)2/2R; where R stands for reflectance). The probe was attached at the top of the quartz microreactor within a distance of 2–3 mm from the catalyst bed of 4–5 mm thickness. 

## 4. Conclusions

Nanoreplication provides the possibility of obtaining thermally stable (up to 700 °C) mesoporous alumina support with ordered pore arrangement and specific surface as high as 270 m^2^/g. Based on this support Cr-containing catalysts for the PDH process can be obtained by impregnation with various amounts of chromium oxide active phase. The optimal propene yield of 32% was reached at 550 °C in the sample containing 20 wt % of Cr_2_O_3_. In the studied catalysts both redox and non-redox Cr species coexist. The concentration of the redox sites in respect to the non-redox ones decreases with the total Cr content. The Cr(V) and Cr(VI) species present in the fresh or regenerated catalysts are the precursors of redox Cr(III) species that are formed in the initial period of the dehydrogenation process. These species seem to be responsible for the catalytic activity at a low Cr content, while at higher Cr loadings both redox and non-redox in origin Cr(III) species influence the catalytic activity. A comparative investigation of chromium oxide-based materials supported on nanocasted and commercial alumina revealed that the former samples had better resistance to deactivation.

## Figures and Tables

**Table 1:** Phase composition and textural properties of SBA-15, CMK-3 and mesoporous supports obtained by nanocasting. *S*_BET_: specific surface area; *V*_micro_: micropore pore volume; *V*_meso_: mesopore volume; *V*_total_: total pore volume.

Sample	Calcination/Pretreatment Temperature (°C)	Phase Composition	*S*_BET_ (m^2^∙g^−1^)	*V*_micro_ (cm^3^∙g^−1^)	*V*_meso_ (cm^3^∙g^−1^)	*V*_total_ (cm^3^∙g^−1^)
SBA-15 ^a^	550	-	756	0.01	0.83	0.84
CMK-3 ^a^	800	-	1411	0.10	1.22	1.23
Al_2_O_3_-n	600	-	259	0.01	0.32	0.33
	700	-	270	0.00	0.41	0.41
	800	*γ*-Al_2_O_3_	254	0.00	0.44	0.44
	900	*γ-*Al_2_O_3_	178	0.02	0.35	0.37

^a^ data from [37].

**Table 2:** Base characterization of Cr*x*/Al_2_O_3_-n catalysts.

Sample	Cr_tot_ Content ^a^ (wt % of Cr_2_O_3_)	H_2_-TPR		NH_3_-TPD (μmol NH_3_∙m^−2^) ^b^			S_BET_ (m^2^∙g^−1^)	V_total_ (cm^3^∙g^−1^)
		H_2_ mmol·g^−1^	H_2_/Cr_tot_	Weak	Medium-Strong	Total		
Cr1/Al_2_O_3_-n	1.2	0.16	1.34	0.26	1.31	1.57	232	0.36
Cr5/Al_2_O_3_-n	5.1	0.67	0.94	0.43	1.93	2.36	175	0.23
Cr10/Al_2_O_3_-n	10.1	1.33	0.54	0.31	2.02	2.33	170	0.12
Cr20/Al_2_O_3_-n	22.5	2.96	0.27	0.63	2.23	2.87	148	0.13
Cr30/Al_2_O_3_-n	28.7	3.78	0.25	0.59	1.31	1.90	129	0.12

^a^ Total Cr content calculated by inductively-coupled plasma (ICP) in wt % of Cr_2_O_3_. ^b^ Number of acid sites estimated based on deconvolution of NH_3_-TPD profiles (Figure S4).

**Table 3:** Initial catalytic performances in dehydrogenation of propane to propene ^a^.

Sample	Temp. (°C)	Conversion (%)	Yield (%)	Selectivity (%)			
		C_3_H_8_	C_3_H_6_	C_3_H_6_	C_2_H_6_	C_2_H_4_	CH_4_
Cr1/Al_2_O_3_-n	550	13.0	10.4	79.9	1.4	12.1	6.6
Cr5/Al_2_O_3_-n	550	21.9	19.4	88.8	2.3	5.1	3.9
Cr10/Al_2_O_3_n	550	24.6	22.8	92.3	1.6	2.9	3.2
Cr20/Al_2_O_3_-n	500	12.9	11.3	87.7	1.8	5.2	5.3
	550	33.8	31.7	94.0	1.4	2.1	2.4
	600	41.8	37.5	89.8	2.3	3.9	3.9
Cr30/Al_2_O_3_-n	550	17.8	17.5	98.2	0.3	0.4	0.2
Cr20/SBA-15	550	25.7	22.1	86.0	4.8	2.7	5.6
Cr20/MCM-41	550	28.9	24.9	86.0	4.0	4.6	5.4
Cr20/CMK-3 ^b^	550	47.4	40.1	84.7	3.3	3.8	8.4

^a^ Reaction conditions: Weight hourly space velocity WHSV = 1.2 h^−1^; C_3_H_8_:He molar ratio = 1:14; Total flow rate = 30 cm^3^∙min^−1^; Catalyst weight = 200 mg; Results are summarized after 10 min-on-stream. ^b^ from [37].

## Supplementary Materials

The following materials are available online at http://www.mdpi.com/2079-4991/7/9/249/s1, S1: Preparation of hard templates; Figure S1: N_2_ adsorption–desorption isotherms of alumina samples calcined at 600, 700, 800 and 900 °C; Figure S2: XRD patterns of alumina samples calcined at 600, 700, 800 and 900 °C; Figure S3: N_2_ adsorption–desorption isotherms of Cr*x*/Al_2_O_3_-n catalysts, Figure S4: NH_3_-TPD profiles of pure Al_2_O_3_-n (calcined at 700 °C) and Cr*x*/Al_2_O_3_-n catalysts; Table S1: Base characterization of Al_2_O_3_-c support; Figure S5: Variation of propane conversion and selectivity to propylene with time-on-stream (TOS) over Cr*x*/Al_2_O_3_-n catalysts with different Cr_2_O_3_ content.
