# Supplementary material for: Catalytic Behavior of Chromium Oxide Supported on Nanocasting-Prepared Mesoporous Alumina in Dehydrogenation of Propane
Source: Nanomaterials (Basel). 2017 Sep 1;7(9):249. doi: 10.3390/nano7090249 (PMC5618360; doi:10.3390/nano7090249)
Supplement: Supplementary file 1 [file nanomaterials-07-00249-s001.pdf]

Catalytic Behavior of Chromium Oxide Supported on Nanocasting-Prepared Mesoporous Alumina in Dehydrogenation of Propane

Adam Węgrzyniak[1], Sebastian Jarczewski[2], Adam Węgrzynowicz[1], Barbara Michorczyk[1], Piotr Kuśtrowski[2], Piotr Michorczyk[1,*]

[1] Institute of Organic Chemistry and Technology, Cracow University of Technology, Warszawska 24, 31-155 Kraków, Poland; wegrzyniak@indy.chemia.pk.edu.pl (A.W.); vinnicki@chemia.pk.edu.pl (A.W.); bmichorczyk@indy.chemia. pk.edu.pl (BM)

[2] Faculty of Chemistry, Jagiellonian University, Gronostajowa 2, 30-387 Kraków, Poland; jarczewski@chemia.*uj*.edu.pl (S.J.); kustrows@chemia.uj.edu.pl (P.K.)

* Correspondence: pmichor@pk.edu.pl, Tel.: +48 12 628-27-51

S1. Preparation of Hard Templates

In typical synthesis of SBA-15, 8 g of Pluronic P123 (EO20PO70EO20, Mav=5800, from Aldrich, U.S.) was dissolved in a solution containing 60 g of distilled water and 120 g of 2 M HCl (Polish Chemical Reagents, Gliwice, Poland) at 35 °C. The mixture was stirred until complete dissolution of Pluronic P123. Next, 17 g of tetraethyl orthosilicate (98%, Aldrich, China) was added dropwise to the transparent solution, while stirring (400 rpm) continued at 35 °C. Finally, the mixture was stirred (400 rpm) at 35 °C for another 20 h and then hydrothermally treated under static conditions at 90 °C for 24 h. The resultant white precipitate was filtered off without washing, dried at 60 °C overnight and calcined in air by increasing the temperature from ambient to 550 °C, over a 9-h period and then maintaining this temperature for another 12 h.

CMK-3 carbon was prepared using SBA-15 as the hard template and an aqueous solution of sucrose as the carbon precursor. For the CMK-3 preparation, mesoporous silica (1 g) was impregnated with a solution containing 5 g of water, 1.25 g of sucrose and 0.14 g of sulfuric acid. The sample was dried in two steps: at 100 °C for 6 h and then at 160 °C for another 6 h. The sample of SBA-15 containing partially polymerized and carbonized sucrose was impregnated again. In the second impregnation, a solution with 0.8 g of sucrose and 0.09 g of sulfuric acid dissolved in 3 g of water was used. The drying procedure was repeated. Carbonization was completed by increasing the temperature (1 °C/min) up to 800 °C in flowing N_2 and by using an isothermal step at 800 °C for another 12 h. The carbon replicas were obtained by treatment of the composite material with 10 wt% HF solution twice. Thermogravimetry analysis by heating CMK-3 after washing to 900 °C in air showed that almost 100% weight loss occurred, demonstrating a complete removal of the silica template.

S2. Alumina support characterization

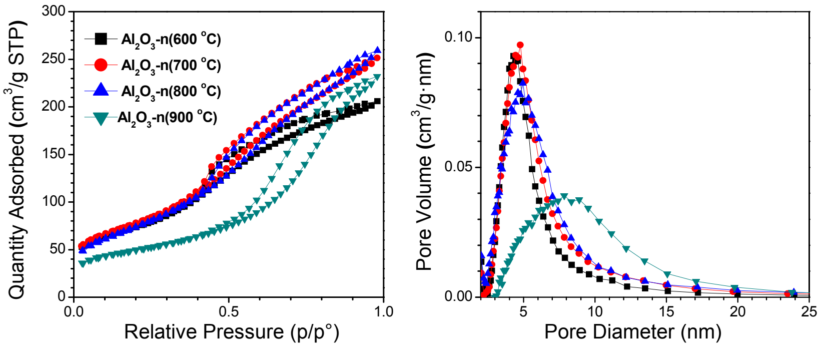

Figure S1. N_2 adsorption–desorption isotherms of alumina samples calcined at 600, 700, 800 and 900 °C.

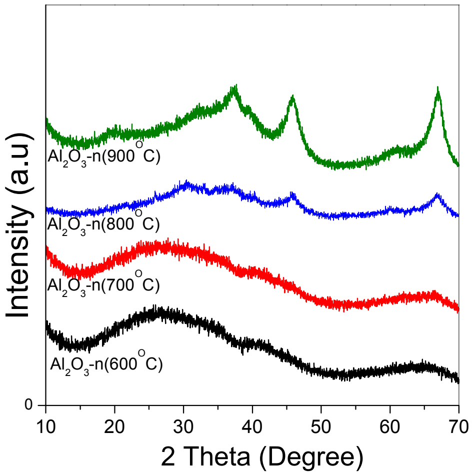

Figure S2. XRD patterns of alumina samples calcined at 600, 700, 800 and 900 °C.

Table S1. Base characterization of Al_2O_3-c.

Phase composition	S_{BET} ($m^2 \cdot g^{-1}$)	V_{total} ($cm^3 \cdot g^{-1}$)	*NH_3-TPD*[a] (*µmol $NH_3 \cdot m^{-2}$*)		
			Low	*Medium–strong*	*Total*
γ- Al_2O_3	161	0.24	1.18	1.43	2.61

[a] Number of acid sites estimated based on deconvolution of the NH_3-TPD profile.

S.3. Catalyst characterization

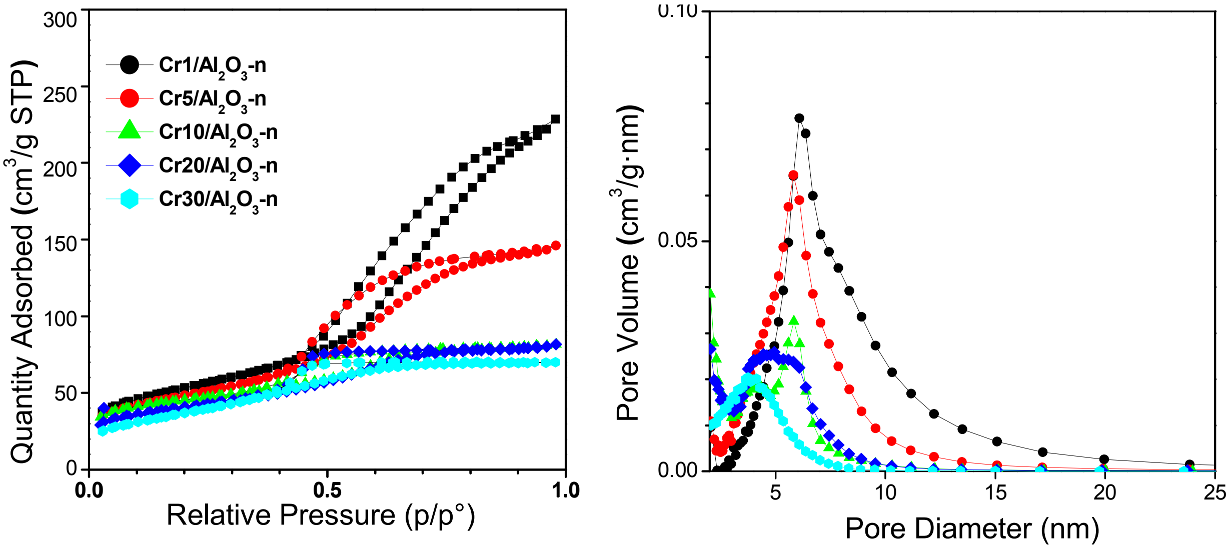

Figure S3. N_2 adsorption-desorption isotherms of Cr*x*/Al_2O_3-n catalysts.

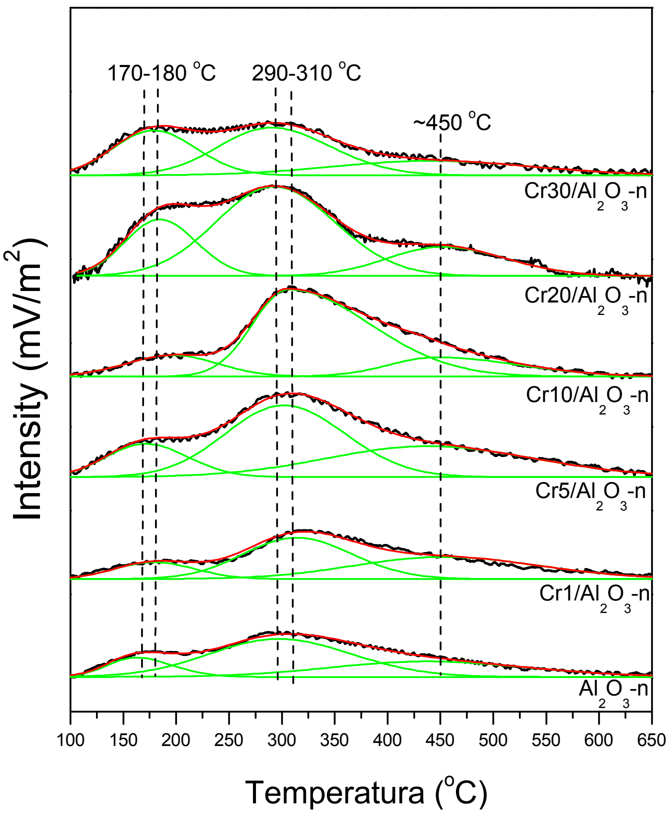

Figure S4. NH_3-TPD profiles of pure Al_2O_3-n (calcined at 700 °C) and Cr*x*/Al_2O_3-n catalysts.

S.4. Catalytic tests

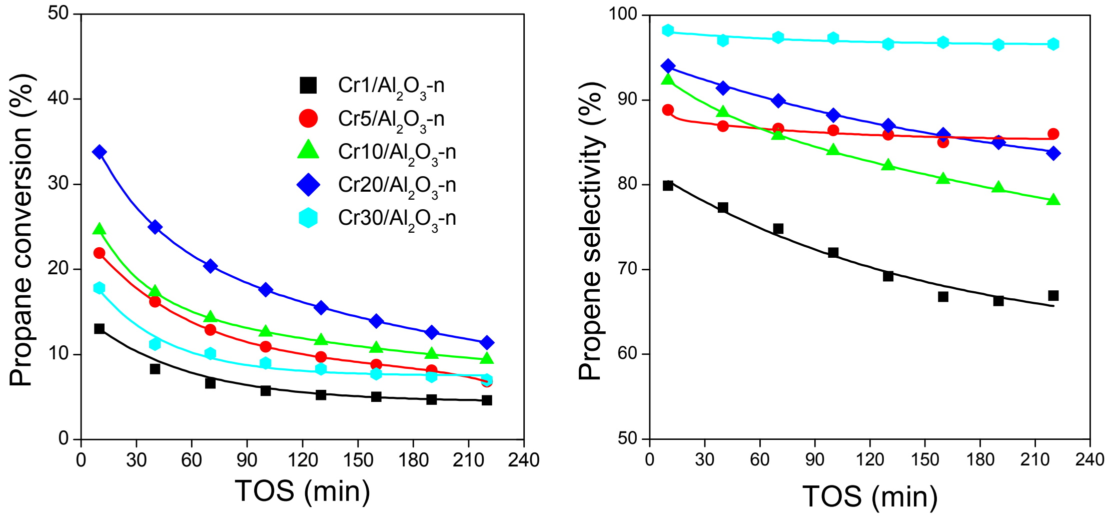

Figure S5. Variation of propane conversion and selectivity to propylene with time-on-stream (TOS) over Cr*x*/Al_2O_3-n catalysts with different Cr_2O_3 content. Dehydrogenation conditions: reaction temperature = 550 °C; catalysts weight = 200 mg; feed gas composition C_3H_8:He = 1:14; total flow rate = 30 $cm^3 \cdot min^{-1}$.
